# Magnitude of delayed turnaround time of laboratory results in Amhara Public Health Institute, Bahir Dar, Ethiopia

**DOI:** 10.1186/s12913-019-4077-2

**Published:** 2019-04-24

**Authors:** Melashu Balew Shiferaw, Gizachew Yismaw

**Affiliations:** Amhara Public Health Institute, P.O.Box 447, Bahir Dar, Amhara Ethiopia

**Keywords:** Delayed TAT, APHI, Laboratory, Ethiopia

## Abstract

**Background:**

Clinical decisions depend on timely laboratory result reporting. The timeliness is commonly expressed in turnaround time and serves as a quality improvement tool to assess the effectiveness and efficiency of the laboratory. According to the International Organization for Standardization (ISO) guidelines, each laboratory shall establish turnaround times for each of its examinations that reflect clinical needs, and shall periodically evaluate whether or not it is meeting the established turnaround times. Therefore, this study aimed to assess the TAT of laboratory results done in the reference laboratories of the Amhara Public Health Institute, Bahir Dar, Ethiopia.

**Methods:**

A retrospective cross sectional study was carried out from 01 January to 31 September 2018. Each patient sample was considered as a study unit. Nine months data were extracted from the sample tracking log and from the Laboratory Information System (LIS) database. Descriptive and summary statistics were calculated using SPSS version 20.0 statistical software.

**Results:**

A total of 34,233 patients samples were tested during the study period. Monthly average TAT ranged from 38.6 to 51.3 days for tuberculosis (TB) culture, 5.3 to 42.4 days for exposed infant diagnosis (EID) for HIV, 8.4 to 26 days for HIV 1 viral load, and 1.9 to 3.5 days for TB genexpert tests. Compared with the standard, 76.5% of the viral load, 68.1% of the EID for HIV and 53.8% of the TB genexpert tests had delayed TAT. Repeated reagent stock out, high workload, activities overlapping, and staff turnover were major reasons for the result delays.

**Conclusions:**

There was a delayed turnaround time of laboratory results in APHI. HIV viral load, EID and TB genexpert results were the most affected tests. Workload reduction plan, proper stock management, specific work assignment and trained staff retention are important approaches to minimize the delayed TAT in the setting.

## Background

Turnaround time (TAT) is defined as the elapsed time between two specified points through pre-examination, examination and post-examination processes of the laboratory testing [1]. It consists of the intervals from order placement to specimen collection, transportation to the laboratory, accessioning in the laboratory, centrifugation, aliquoting, additional pre-analytic steps if necessary, transport times within and between laboratories, analysis time, the time after completion of analysis until result verification, and the time it takes for the clinical team to be informed of the result [2].

In order to ensure quality diagnostic service, the laboratory should provide accurate, reliable and timely results to the customers. The timeliness is commonly expressed in TAT, which is often used by the clinicians as the benchmark for laboratory performance, and serves as a quality indicator to assess the effectiveness and efficiency of the total testing process in addition to clinicians’ and patients’ satisfaction [3–6].

Assessment and improving turnaround time is essential for laboratory quality management [2]. According to the International Organization for Standardization (ISO) guidelines, the laboratory, in consultation with the users, shall establish turnaround times for each of its examinations that reflect clinical needs. The laboratory shall periodically evaluate whether or not it is meeting the established turnaround times [1]. The laboratory plays a crucial role to provide objective information. In real practice, 60 to 70% of the objective information on the patient’s chart is laboratory information [7].

Delays in laboratory results reporting would cause a delay in the diagnosis and management of patients. A study showed that there was 43% treatment delay and 61% increased length of stay in the emergency department [8]. Moreover, a slow TAT can lead to increase in requests which results in duplication of the test [9]. This further increases the workload in the laboratory, and may again increase the cost burden of the health care [10].Therefore, faster TAT is universally desirable for efficient and effective management of patients in addition to save time and money [8].

When there is a delay, understanding the root causes of high TAT using evidence-based methods are essential for quality improvements. Consequently, regulatory and accrediting bodies advise clinical laboratories to target TAT in their continual improvement process [8, 11]. However, information about the problem is limited in the region including our setting. Therefore, this study was designed to assess the magnitude of delayed TAT of laboratory tests in Amhara Public Health Institute, Bahir Dar, Ethiopia.

## Methods

A retrospective cross sectional study was carried out to assess the trends of TAT of laboratory tests performed in the reference laboratories of APHI. The institute is located in Bahir Dar town Ethiopia to serve as a reference laboratory for peripheral laboratories in Amhara region in addition to providing active patient laboratory diagnostic service provision. The institute has seven reference laboratories namely HIV molecular, measles/rubella, immunology and hematology, clinical chemistry, parasitology, basic microbiology and TB reference laboratories.

**Fig. 1:**
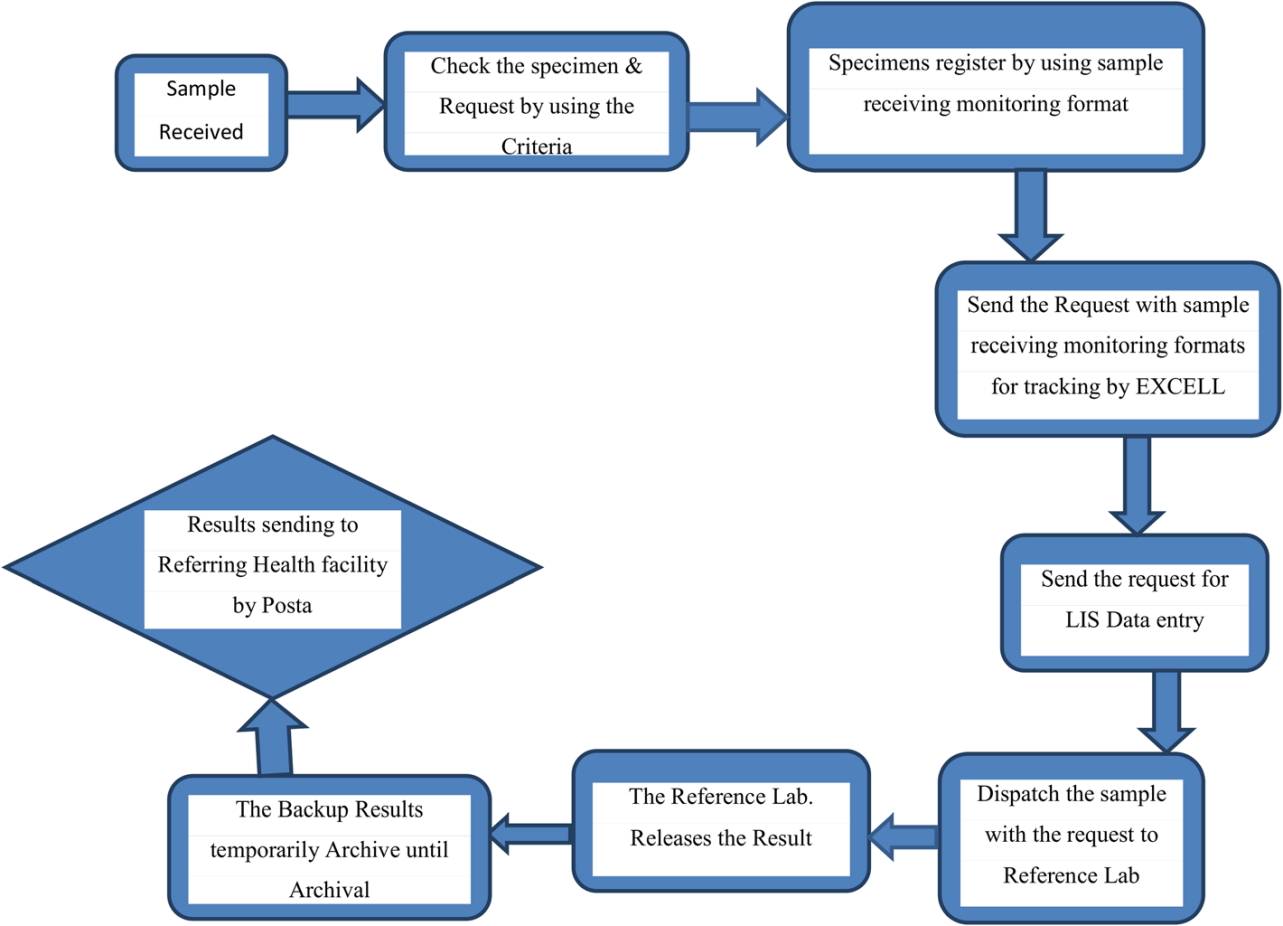
Process of work flow for laboratory diagnosis in the reference laboratories of the APHI, 2018. Abbreviations: LIS, laboratory information system; Lab, laboratory

The patients who had final released results at APHI central reception were included in this study. Therefore, each sample was considered as a sampling unit to study the TAT of the test. As a requirement, the reference laboratories established specific TAT of each test to follow as a quality indicator. The laboratories again established a target for each test. For instance, EID and HIV viral load tests had a target TAT of 10 days each. TB genexpert and clinical chemistry tests had also targeted TAT of two hours each. As a quality indicator of good performance, at least 90% of the tests should be released within the established TAT from the central reception. In APHI all patient samples were submitted at the central reception, evaluated based on established criteria, entered to the Polytech Laboratory Information System (Comp Pro Med, Inc., USA) and samples dispatched to each respective laboratories, and then results sent back to central reception after investigation and result verification (Fig. 1). In addition, referral samples collected at different health facilities were transported to the APHI through postal and/or vehicle dedicated to the specimen transport. All specimens transported and submitted to the APHI reception, and after laboratory analysis results were back to the health facilities through postal services.

All submitted samples at APHI reception with complete information (from 01 January to 31 September 2018) were included consecutively. In this study, data extraction tool was prepared by the principal investigator. The tool was pretested and modifications were made after the pretest to ensure the quality of the data extraction tool. In the central reception, information about received samples was available from the specimen tracking log. The tracking log was reviewed and necessary data related to TAT were collected from the tracking log and from the Polytech Laboratory Information System (Comp Pro Med, Inc., USA) database. Then, TAT for each submitted specimen having final results was calculated by subtracting the time of specimen submission from the time of result released at the APHI central reception. This TAT did not include travel to the testing laboratory. The TAT of each patient result was graded as good if it was released within the time frame established by the APHI laboratories. Moreover, the general performance of the laboratory was rated as good if at least 90% of the patient results were released within the established TAT on a monthly basis.

We also interviewed laboratory personnel who worked in the reference laboratories and in the central receptions of the APHI to describe the causes of delayed TAT. Reagent stock status, activities overlapping, equipment status and workload statistics with number of laboratory personnel were the main focus of the interviews in addition to document review.

The data were analyzed using SPSS 20.0 (IBM Corp., Armonk, NY); cleaning analysis of missing values were checked; variables such as type of laboratory tests done in APHI, date and time of specimen processing, days of reagent stock out, overlapping of other activities and workload were analysed.TAT indicators such as proportions and mean were calculated [12, 13].

## Results

A total of 34,233 samples were diagnosed in the APHI reference laboratories during the nine month period, January to September 2018. HIV viral load and leishmania diagnosis showed the highest and the lowest number of tests done with 26,984 and 21 total testing, respectively. Moreover, a total of 2484 immune hematology, 1393 EID and 1236 TB genexpert tests were performed. The workload was improved time to time. However, clinical chemistry laboratory had a high workload in May 2018 due to sudden increase in test volumes. Measles, basic microbiological culture and sensitivity, TB culture and chemistry accounted 320, 421, 946 and 428 test volumes, respectively. The highest test statistics across the months were 5497, which was recorded in March and the lowest test statistics, 2636, was recorded in September 2018 (Table 1).

**Table 1:** Number of laboratory tests performed at APHI from January to September, 2018

SN	Test type	Number of tests									Total
		Jan	Feb	Mar	Apr	May	Jun	Jul	Aug	Sep	
1	HIV Viral load	3725	2497	4192	4057	2637	2526	2405	2719	2226	26,984
2	EID	403	135	217	74	165	37	163	146	53	1393
3	MDR TB culture	112	97	125	78	64	98	127	98	147	946
4	Genexpert	190	197	186	230	161	109	63	28	72	1236
5	Basic culture and sensitivity	15	21	231	10	14	68	13	35	14	421
6	Clinical chemistry	10	23	34	4	316	11	8	15	7	428
7	Leishmania	2	7	3	1	0	3	1	1	3	21
8	Measles	33	36	62	46	84	46	13	0	0	320
9	Immunohematology	393	451	447	278	382	222	42	155	114	2484
Total		4883	3464	5497	4778	3823	3120	2835	3197	2636	34,233

Abbreviations: *EID* exposed infant diagnosis, *HIV* human immunodeficiency virus, *TB* tuberculosis

The average TAT of each test varied across each test parameter. Over the nine month period, the average TAT for HIV viral load was 26 days in February and 8.4 days in May 2018. EID showed greater differences with a maximum of 42.4 and a minimum of 5.3 days to provide patient results. The fastest TAT was documented for immunohematoloy and clinical chemistry tests that were done in two hours. The average time taken for genexpert was between 1.9 and 3.5 days. TB culture took more time due to the nature of the TB bacteria that wasted from 38.6 to 51.3 days to get final patient results. Looking at these trends, most of the tests had improved average monthly TAT in April, May, June and July 2018 (Table 2).

**Table 2:** Trends of average TAT of laboratory tests in APHI from January to September 2018

SN	Test type		Target TAT	TAT								
				Jan	Feb	Mar	Apr	May	Jun	Jul	Aug	Sep
1	Viral load		10 days	26.1	19	21	11.4	8.4	9.9	15	13.8	12
2	EID		10 days	42.4	20.3	11	12.5	13	5.3	19	27	11
3	MDR TB		64 days	51.3	44.7	51.1	44	38.6	46	45	45.6	48
4	Genexpert		2 days	2.69	3	2.4	3.5	2.4	2.2	3.3	1.9	2.4
5	Basic culture and sensitivity	Blood	8 days	8	7	7	7	7	7	7	7	7
		Others	3 days	2.3	2.8	2. 5	3	3. 3	3	3. 5	3. 5	3. 5
6	Clinical chemistry		2 h	2	2	20.3	10	9.15	2.2	1.5	1.5	2.4
7	Measles		7 days	21	9.9	9	7.7	10.7	7	7	7	7
8	Immunohematology		1 h	2.5	2.3	1.84	3.4	14.1	18.0	21	14. 3	17.6

Abbreviations: *EID* exposed infant diagnosis, *HIV* human immunodeficiency virus, *MDR* multidrug resistance, *TB* tuberculosis

Other cultures: Pus, urine, stool, genital swab, ear swab and throat

**Fig. 2:**
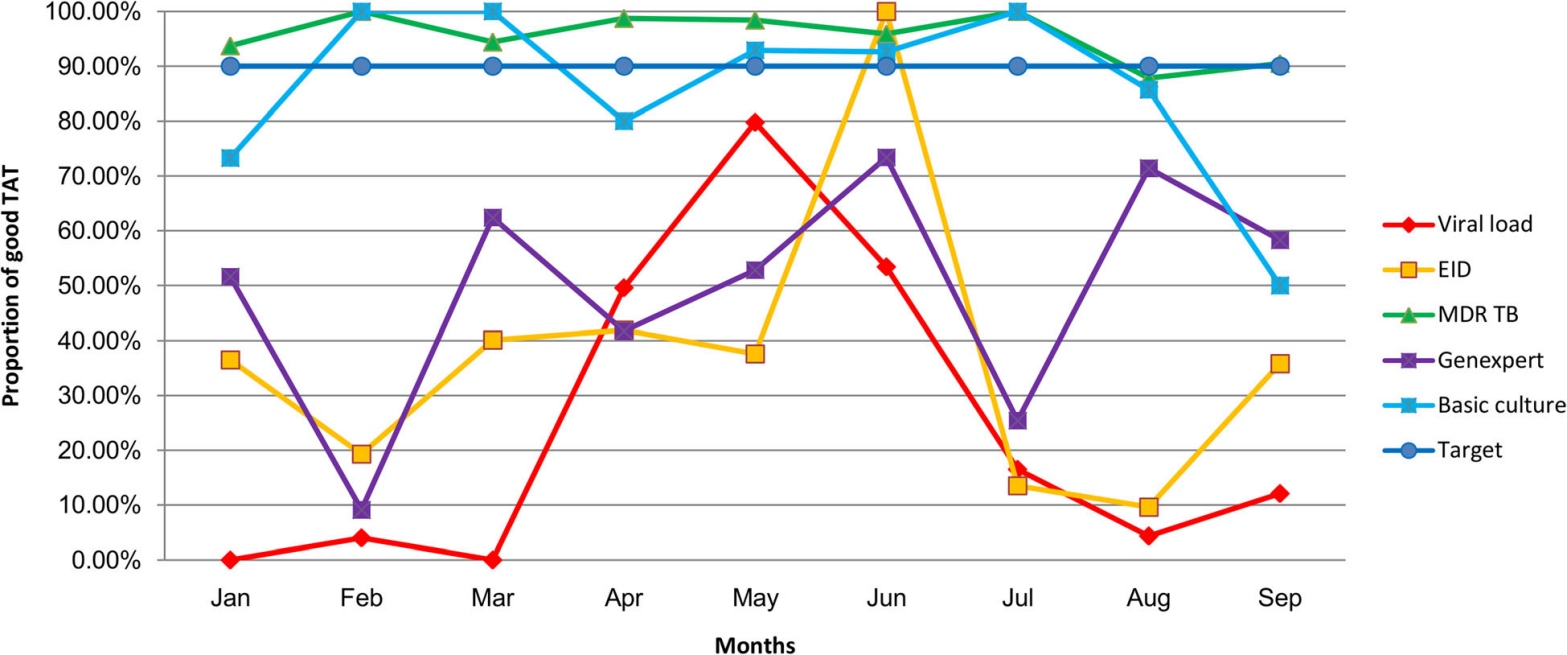
Trends of laboratory results released based on the established TAT target of APHI from January to September 2018. Abbreviations: EID exposed infant diagnosis; TB, tuberculosis; MDR, multidrug resistance; VL, viral load

Among the total tests requested in the APHI reference laboratories, HIV viral load, EID and TB genexpert test results were out of the established 90% target over the nine months. About 76.5% (20,634/26984) of viral load, 53.8% (665/1236) of TB genexpert and 68.1% (984/1393) of EID results had delayed TAT in the study period. EID TAT was out of the target for eight months follow up. Interestingly, in May 2018, all of the EID samples were analyzed within the established TAT target. Similarly, TB culture was also achieved above the target except in August 2018 when only 87.8% of the TB culture results were released within the established TAT. The TAT of basic culture and sensitivity tests were out of the established TAT in January, April, August and September. In between the TATs improved. However, it has become delayed since August 2018(Fig. 2) as a result of staff turnover. Other tests (Leishmania culture, measles, clinical chemistry and immunohematology) results were released within the established laboratory targeted TAT.

During the study period, there were repeated stock outs of both viral load and EID reagents specifically happened. In February and April 2018, both of the tests lasted for14 days of stock out. Equipment downtime, and low equipment capacity to perform high number of samples were also challenges in the institute. Moreover, the number of personnel assigned to testing was lower compared to the workload statistics in addition to overlapping of other activities in the testing laboratory.

## Discussion

Appropriate and timely clinical decisions depend on timely reporting, which in turn affects patient outcome. Hence, a rapid laboratory turnaround time is important to manage patients in a timely manner. So, analysis of this time interval helps to determine the cause of delay, which is then followed by the improvement in turnaround time [14]. In the present study, the turnaround time of laboratory tests done in APHI over the nine month period was analyzed. Among the total tests, viral load, EID and TB genexpert had delayed TAT.

In this study, HIV 1 viral load tests took an average of about 15 days (minimum 8.4 and maximum 26 days) over the period of nine months. According to the APHI laboratory policy, at least 90% of the viral load results should have been released within 10 days. However, only 23.5% (6350/26984) of the routine viral load results were released within the established TAT. This might be due to the high workload that our finding showed a total of viral load testing was 78.8% of the total 34,233 laboratory tests done in APHI. In addition, there was repeated stock out of viral load reagents that occurred in the HIV molecular laboratory during the study period. There was also activity overlaps such as supportive supervision, mentorship, and extensive provision of trainings to peripheral laboratories making the laboratory personnel busier to do such activities that could delay the TAT of patient results. The most common reason for the laboratory result delay was noted by other studies and found to be a machine breakdown followed by problems in reagent stock out, machine maintenance and overlook of technical staff, and having increased number of test menus [15, 16]. A study conducted in Malawi also showed longer TAT of viral load testing was mainly linked with specimen origin, type and testing laboratory [17]. Delayed TATs could greatly impact early management and care of the patients. Specifically, it could delay initiation of treatment adherence counseling and/or switch to second line antiretroviral therapy (ART) in patients experiencing treatment failure, which further contributes to poorer health outcomes, prolonged immune activation, development of drug resistance, and increased mortality [18–21]. The problem could be solved by ensuring a consistent supply and placement of enough manpower in addition to planning of overlapped activities in the institute laboratories. This would be helpful in providing better service to the physicians and patients.

Moreover, 68.1% of the EID results had delayed TAT over the nine month period. It took 17.9 days on average with a range of 5.3 to 42.4 days. Although the average trend of TAT became continuously improved from January to June 2018, it was again increased since July. This finding was relatively better compared to a study done in Kenya that reported an average TAT of 24.7 days for EID services [22]. However, compared to the established TAT, still it needs more improvement to accomplish the target of the APHI laboratory. The APHI laboratory established 10 days target and at least 90% of the results to be released within the target TAT like that of the viral load testing. Documents revealed that very early infant diagnosis, defined as testing within two weeks of life, combined with rapid ART initiation could prevent the observed decline in immunologic function and clinical deterioration and further reduce infant mortality [23].

Currently, priority is given more to tuberculosis prevention and control. One of the strategies is early diagnosis and treatment. In the institute, TB culture and genexpert tests have been given with the aim to deliver results in 15 to 48 for positive and 42–64 days for negative cultures, and 2 days for genexpert testing. Although the average TAT of TB culture seemed long time (38.6 to 51.3 days), it was due to the nature of the testing that TB bacteria need more time to grow. In a pilot study, the TAT of the Xpert MTB/RIF assay in an outpatient setting was shorter than those of AFB smears, liquid culture, solid culture and drug sensitivity tests in terms of the interval of reporting the result from the laboratory, as well as interval to the confirmation of results by physicians. In particular, the Xpert MTB/RIF assay shortened the time to initiation of anti-TB drugs by 14 days [24]. However, in this study, 53.8% of TB genexpert tests were out of the established TAT. This needs close follow up and monitoring to improve the Xpert MTB/RIF assay diagnostic service more.

As a limitation, this study was carried out using data from secondary sources that might underestimate the true delays of laboratory turnaround times and may also missed required information.

## Conclusions

There was a delayed turnaround time of laboratory results in APHI. HIV viral load, EID and TB genexpert results were the most affected tests that need improvement. Workload reduction plan, proper stock management, specific work assignment and trained staff retention are important approaches to minimize the delayed TAT in the setting. Moreover, we recommend strong prospective studies to improve the laboratory services more.

## Abbreviations

APHI: Amhara Public Health Institute
ART: Antiretroviral Therapy
EID: Exposed Infant Diagnosis
ISO: International Organization for Standardization
LIS: Laboratory Information System
TAT: Turnaround Time
TB: Tuberculosis
